# Clinical Presentation, Diagnosis, and Genetic Insights of Miyoshi Myopathy: A Case Report and Literature Review

**DOI:** 10.7759/cureus.68869

**Published:** 2024-09-07

**Authors:** Łukasz Stolarski, Patryk Patrzałek, Friederike Gerber, Wojciech Tokarczyk, Maksymilian Bialasik-Misiorny, Marek Kulma

**Affiliations:** 1 Intensive Care Unit, District Hospital in Rawicz, Rawicz, POL; 2 Surgery, District Hospital in Rawicz, Rawicz, POL; 3 Internal Medicine, Wroclaw Medical University, Wrocław, POL; 4 Cardiology, Wrocław University Hospital, Wrocław, POL; 5 Cardiology, Poznań University of Medical Sciences, Poznań, POL; 6 Neurology, Marek Kulma Praktyka Lekarska, Gorzkowice, POL

**Keywords:** axonal damage, dysferlinopathy, dysf gene, genetic muscle disorders, genetic test, lower limb weakness, miyoshi myopathy

## Abstract

Miyoshi myopathy (MM) is an autosomal recessive dysferlinopathy caused by a mutation in the dysferlin (DYSF) gene on chromosome 2p. Recent findings indicate that MM and Lower Girdle Muscular Dystrophy R2 (LGMD2B) are the same disease.

We present the case of a 44-year-old male who first experienced symptoms of MM at the age of 19, initially noticing difficulty climbing stairs and standing on his toes. By the age of 29, he had developed significant calf muscle atrophy and weakness, which led to difficulties with walking. Electromyography and nerve conduction studies showed axonal damage and myogenic features. Genetic testing ruled out Charcot-Marie-Tooth disease but identified a pathogenic variant in the DYSF gene.

Laboratory tests revealed elevated creatine kinase levels. Photographs of the patient's lower limbs showed significant calf muscle atrophy. Based on clinical, laboratory, and electrophysiological findings, he was diagnosed with MM. This case highlights the importance of genetic testing in diagnosing muscular dystrophies and underscores the need for continued research into gene and cell therapies.

To the best of our knowledge, this is one of the first studies reporting a case of MM in Poland.

## Introduction

Miyoshi myopathy (MM) is an inherited autosomal recessive dysferlinopathy caused by a mutation in the dysferlin (DYSF) gene, located on chromosome 2p, which encodes dysferlin, a protein involved in muscle membrane repair [1].

The first cases of MM were observed and studied in Japan by Miyoshi K et al. in 1977 [2]. Symptoms often begin in early adulthood, typically starting in the distal lower limbs, especially in the posterior calf muscles. As the condition progresses, individuals may require support for walking and eventually may become bedridden [3].

A characteristic feature of the disease is a significantly elevated serum creatine kinase (CK) level, often reaching 20 to 150 times the normal upper limit [4].

Diagnostic procedures include genetic testing to confirm the presence of homozygous or compound heterozygous mutations in the DYSF gene [5].

Due to its rarity and the similarity of its symptoms to other muscular dystrophies and neuropathies, MM often remains undiagnosed or is misdiagnosed. Early recognition and accurate diagnosis are crucial for ensuring appropriate treatment and improving the quality of life for patients [6]. This case highlights the importance of increased awareness and clinical vigilance in distinguishing MM from other neuromuscular disorders, thereby facilitating timely intervention.

To the best of our knowledge, this is one of the first documented cases of a Polish patient diagnosed with MM.

## Case presentation

We present the case of a 44-year-old Caucasian male whose initial symptoms began at the age of 19. During military training, he noticed difficulties with jumping, experiencing a sensation of weights attached to his feet, making it hard to lift them off the ground. Additionally, at night, he suffered from intense burning sensations in his feet, which were alleviated by elevating his legs. By the age of 29, the patient observed significant muscle weakness in the lower limbs, particularly in the calves, along with noticeable muscle atrophy. He had trouble climbing stairs, requiring support from the handrail. By the age of 34, he developed walking difficulties, characterized by foot dragging, and two years later, experienced upper limb weakness primarily affecting the arm muscles. Similar symptoms have appeared in the patient’s younger sister, although she has not been diagnosed at this time.

In the neurological examination, no abnormalities were observed in the cranial nerves. The patient presented with symmetrical global weakness of the lower limbs: hip flexion 4/5 on the Modified Medical Research Council (mMRC) scale, extension 4-/5 on the mMRC scale, knee flexion 3/5 on the mMRC scale, extension 4/5 on the mMRC scale, dorsiflexion of the feet 2/5 on the mMRC scale, and plantar flexion 3/5 on the mMRC scale. Deep tendon reflexes were symmetrically diminished. Notably, there was significant bilateral atrophy of the posterior calf muscles, predominantly affecting the gastrocnemius muscle. Sensory examination revealed no deficits in superficial or deep sensation, and no signs of ataxia were present. The Romberg test was performed and yielded normal results. The patient's gait was characterized by a waddling motion with a steppage component.

Nerve conduction studies (NCS) revealed axonal damage to the motor fibers of the right peroneal nerve, along with F-wave abnormalities indicating proximal nerve damage. Conduction in the motor fibers of the right tibial nerve and sensory fibers of the right sural nerve remained normal. Electromyography (EMG) revealed significant alterations in muscle activity, particularly in the right anterior tibial muscle and the right rectus femoris muscle. In the right anterior tibial muscle, electrical silence was observed at rest, while the effort recording was rich, with pathological interference and reduced amplitude. Additionally, the amplitude of the single motor unit potential was decreased, and the duration of this potential was shortened, although the percentage of polyphasic potentials remained within normal limits. Similarly, in the right rectus femoris muscle, electrical silence was noted at rest, with a rich effort recording that exhibited disturbed gradation of effort. The amplitude was normal but at the lower limit of normal, and the duration of the single motor unit potential was also shortened, with the percentage of polyphasic potentials within normal limits. These findings indicate the presence of pathological changes in the examined muscles, which may suggest neuromuscular damage or another disease process affecting muscle function (Table 1).

**Table 1:** Results of the first performed EMG/NCS. NR: Nerve Recorded; Peak (ms): Peak Latency (in milliseconds); Norm Peak (ms): Normal Peak Latency (in milliseconds); P-T Amp (μV): Peak-to-Trough Amplitude (in microvolts); Norm P-T Amp (μV): Normal Peak-to-Trough Amplitude (in microvolts); O-P Amp (μV): Onset-to-Peak Amplitude (in microvolts); Norm O-P Amp (μV): Normal Onset-to-Peak Amplitude (in microvolts); Delta-0 (ms): Change in Onset Latency (in milliseconds); Dist (cm): Distance (in centimeters); Vel (m/s): Conduction Velocity (in meters per second); Norm Vel (m/s): Normal Conduction Velocity (in meters per second); N: Nerve; R: Root; Onset (ms): Onset Latency (in milliseconds); Norm Onset (ms): Normal Onset Latency (in milliseconds); F-Lat (ms): F Wave Latency (in milliseconds); Lat Norm (ms): Latency Normal (in milliseconds); L-R F-Lat (ms): Left-Right F Wave Latency (in milliseconds); L-R Lat Norm: Left-Right Latency Normal; Ins Act: Insertional Activity; Fibs: Fibrillation Potentials; Psw: Positive Sharp Waves; Amp: Amplitude (in millivolts); Dur: Duration (in milliseconds); Poly: Polyphasic Potentials; Recrt: Recruitment; Int Pat: Interference Pattern; Nml: Normal; Run: Run Number; Rise (ms): Rise Time (in milliseconds); Amp (μV): Amplitude (in microvolts); Dur (ms): Duration (in milliseconds); Area (nVs): Area under the Curve (in nanoVolt-seconds); Phases: Number of Phases; Turns: Number of Turns; Thickness (mV): Amplitude of Turns (in millivolts); Size Index: Size Index (calculated metric); Traces Stored: Indicates if Traces are Stored; Avg Trace Stored: Indicates if the Average Trace is Stored; EMG: Electromyography; NCS: Nerve Conduction Studies.

Nerve Conduction Studies											
Anti-sensory summary table											
Site	NR	Peak (ms)	Norm Peak (ms)	P-T Amp (μV)	Norm O-P Amp	Site 1	Site 2	Delta-0 (ms)	Dist (cm)	Vel (m/s)	Norm Vel (m/s)
Right Sural Anti Sensory (Lat Mall) 25,4°C											
Calf		4.1	<4.0	10.2	>5.0	Calf	Lat Mall	3.4	17.0	50.0	>35
Motor Summary Table											
Site	NR	Onset (ms)	Norm Onset (ms)	O-P Amp (mV)	Norm O-P Amp	Site1	Site2	Delta-0 (ms)	Dist (cm)	Vel (m/s)	Norm Vel (m/s)
Right Peroneal Motor (Ext Dig Brev) 26.1°C											
Ankle		4.4	<6.1	1.3	>2.5	B Fib	Ankle	6.2	34.0	54.8	>38
B Fib		10.6		1.3		Poplt	B Fib	1.6	8.0	50.0	>40
Poplt		12.2		1.3							
Right Tibial Motor (Abd Hall Brev) 25.6°C											
Ankle		3.0	<6.1	13.2	>3.0	Knee	Ankle	8.1	42.0	51.9	>35
F Wave Studies											
NR	F-Lat (ms)	Lat Norm (ms)	L-R F-Lat (ms)	L-R Lat Norm							
Right Peroneal (Mrkrs) (EDB) 26°C											
		<60		<5.1							
Right Tibial (Mrkrs) (Abd Hallucis) 25.4°C											
	46.32	<61		<5.7							
EMG											
Side	Muscle	Nerve	Root	Ins Act	Fibs	Psw	Amp	Dur	Poly	Recrt	Int Pat
Right	AntTibialis	Dp Br Peron	L4-5	Nml	Nml	Nml	Nml	Nml	0	Nml	Nml
Right	RectFemoris	Femoral	L2-4	Nml	Nml	Nml	Nml	Nml	0	Nml	Nml
SMUA Table – (Right AntTibialis)											
Run	Rise (ms)	Amp (μV)	Dur (ms)	Area (mVms)	Phases	Turns	Thickness	Size Index	Traces Stored	Avg Trace Stored	
1	0.63	179.31	3.52	0.14	3	1	0.75	-0.74	Yes	Yes	
2	1.09	256.43	5.23	0.24	3	1	0.95	-0.23	Yes	Yes	
3	0.78	257.92	6.56	0.28	2	2	1.10	-0.08	Yes	Yes	
4	0.86	224.01	7.19	0.22	2	2	0.98	-0.32	Yes	Yes	
5	0.31	356.42	3.13	0.28	2	4	0.79	-0.11	Yes	Yes	
6	0.31	293.71	3.83	0.26	2	2	0.87	-0.19	Yes	Yes	
7	0.86	244.64	5.63	0.21	3	2	0.85	-0.37	Yes	Yes	
8	0.39	164.95	4.3	0.10	2	2	0.62	-0.95	Yes	Yes	
9	0.55	58.10	3.75	0.09	1	0	1.50	-0.97	Yes	Yes	
10	0.86	40.87	3.67	0.09	1	0	2.15	-0.63	Yes	Yes	
11	0.47	73.18	5.08	0.07	1	1	0.96	-1.31	Yes	Yes	
12	0.31	247.95	4.38	0.11	2	2	0.46	-0.75	Yes	Yes	
13	0.86	94.79	6.09	0.12	1	1	1.27	-0.78	Yes	Yes	
14	0.23	142.71	3.36	0.07	2	2	0.51	-1.18	Yes	Yes	
15	0.86	147.80	12.03	0.43	1	1	2.93	1.26	Yes	Yes	
Mean	0.63	185.52	5.18	0.18	1.87	1.53	1.11	-0.49			
StdDev	0.27	93.27	2.26	0.10	0.74	0.99	0.65	0.62			
SMUA Table – (Right RectFemoris)											
Run	Rise (ms)	Amp (μV)	Dur (ms)	Area (mVms)	Phases	Turns	Thickness	Size Index	Traces Stored	Avg Trace Stored	
1	0.94	32.94	4.69	0.05	1	0	1.62	-1.35	Yes	Yes	
2	1.33	149.56	6.09	0.23	2	1	1.51	-0.14	Yes	Yes	
3	1.02	63.98	8.36	0.18	1	1	2.79	0.40	Yes	Yes	
4	0.78	44.96	7.97	0.20	1	1	4.34	1.65	Yes	Yes	
5	1.17	82.40	4.45	0.13	1	0	1.56	-0.60	Yes	Yes	
6	0.70	302.49	10.78	0.66	2	2	2.18	1.14	Yes	Yes	
7	0.47	818.80	4.14	0.79	2	2	0.96	0.79	Yes	Yes	
8	0.39	837.24	6.56	0.51	5	5	0.61	0.46	Yes	Yes	
9	0.31	429.93	5.63	0.33	2	2	0.78	0.04	Yes	Yes	
10	0.23	463.83	4.38	0.56	3	2	1.20	0.53	Yes	Yes	
11	0.63	583.76	6.41	0.61	4	3	1.05	0.58	Yes	Yes	
12	0.86	446.48	7.03	0.71	3	2	1.60	0.90	Yes	Yes	
13	0.23	1440.07	8.52	1.13	4	7	0.79	1.10	Yes	Yes	
14	1.41	464.89	8.13	0.67	4	3	1.33	0.78	Yes	Yes	
Mean	0.75	440.09	6.65	0.48	2.50	2.21	1.60	0.45			
StdDev	0.39	393.97	1.94	0.31	1.34	1.89	0.98	0.77			

Subsequent NCS studies, conducted three years later, indicated progressive, massive axonal damage to the motor fibers of both peroneal nerves (Table 2).

**Table 2:** Follow-up NCS performed three years later. NR: Nerve Recorded; Peak (ms): Peak Latency (in milliseconds); Norm Peak (ms): Normal Peak Latency (in milliseconds); P-T Amp (μV): Peak-to-Trough Amplitude (in microvolts); Norm P-T Amp (μV): Normal Peak-to-Trough Amplitude (in microvolts); O-P Amp (μV): Onset-to-Peak Amplitude (in microvolts); Norm O-P Amp (μV): Normal Onset-to-Peak Amplitude (in microvolts); Delta-0 (ms): Change in Onset Latency (in milliseconds); Dist (cm): Distance (in centimeters); Vel (m/s): Conduction Velocity (in meters per second); Norm Vel (m/s): Normal Conduction Velocity (in meters per second); N: Nerve; R: Root; Onset (ms): Onset Latency (in milliseconds); Norm Onset (ms): Normal Onset Latency (in milliseconds); F-Lat (ms): F Wave Latency (in milliseconds); Lat Norm (ms): Latency Normal (in milliseconds); L-R F-Lat (ms): Left-Right F Wave Latency (in milliseconds); L-R Lat Norm: Left-Right Latency Normal; NCS: Nerve Conduction Studies.

Nerve Conduction Studies											
Anti-Sensory Summary Table											
Site	NR	Peak (ms)	Norm Peak (ms)	P-T Amp (μV)	Norm O-P Amp	Site1	Site2	Delta-0 (ms)	Dist (cm)	Vel (m/s)	Norm Vel (m/s)
Right Median Anti Sensory (2^nd^ Digit) 22.3°C											
Wrist		3.1	<3.6	69.9	>10.0	Wrist	2^nd^ Digit	2.6	14.8	56.9	>39
Right Sural Anti Sensory (Lat Mall) 22.3 °C											
Calf		5.2	<4.0	9.6	>5.0	Calf	Lat Mall	4.1	16.5	40.2	>35
Motor Summary Table											
Site	NR	Onset (ms)	Norm Onset (ms)	O-P Amp (mV)	Norm O-P Amp	Site1	Site2	Delta-0 (ms)	Dist (cm)	Vel (m/s)	Norm Vel (m/s)
Right Median Motor (Abd Poll Brev) 22.3°C											
Wrist		3.8	<4.2	9.6	>5.0	Elbow	Wrist	3.4	22.5	66.2	>50
Elbow		7.2		6.2		Axilla	Elbow	1.6	9.5	59.4	
Axilla		8.8		6.1							
Left Peroneal Motor (Ext Dig Brev) 22.2°C											
Ankle		5.1	<6.1	0.8	>2.5	B Fib	Ankle	7.7	33.5	43.5	>38
B Fib		12.8		0.6		Poplt	B Fib	1.5	6.5	43.3	>40
Poplt		14.3		0.6							
Right Peroneal Motor (Ext Dig Brev) 22.1°C											
Ankle		6.3	<6.1	0.9	>2.5	B Fib	Ankle	8.0	34.0	42.5	>38
B Fib		14.3		0.8		Poplt	B Fib	2.0	8.0	40.0	>40
Poplt		16.3		0.8							
Right Tibial Motor (Abd Hall Brev) 22°C											
Ankle		5.2	<6.1	6.2	>3.0	Knee	Ankle	10.2	41.0	40.2	>35
Knee		15.4		3.4							
F Wave Studies											
NR	F-Lat (ms)	Lat Norm (ms)	L-R F-Lat (ms)	L-R Lat Norm							
Right Median (Mrkrs) (Abd Poll Brev) 22.4°C											
	26.43	<33		<2.2							
Right Tibial (Mrkrs) (Abd Hallucis) 22°C											
	52.87	<61		<5.7							

Considering the overall clinical picture, suspicion of Charcot-Marie-Tooth (CMT) disease was raised. Consequently, a genetic test (MLPA analysis for PMP22 gene duplication) was performed to investigate CMT, which ruled out this diagnosis.

In the interim, the patient underwent a private genetic test for neuromuscular diseases. Whole exome sequencing (WES) via next-generation sequencing (NGS) identified the presence of a known pathogenic variant c.863dupA p.(Asp288GlufsTer40) in both alleles of the DYSF gene in a homozygous state (Table 3).

**Table 3:** Results of the whole exome sequencing. cDNA Notation: Complementary DNA notation describing the specific mutation at the DNA level. Protein Notation: Notation describing the mutation's impact at the protein level. Zygosity: Indicates whether the variant is homozygous (hom.) or heterozygous (het.). Genotype Notation According to HGVS v.15.11 (cDNA): Describes the mutation using the Human Genome Variation Society (HGVS) nomenclature. Allele Frequency in gnomAD (%): Frequency of the variant in the Genome Aggregation Database (gnomAD) expressed as a percentage. Allele Frequency in POLGENOM: Frequency of the variant in the POLGENOM database (if available). ACMG Classification: Classification of the variant according to the American College of Medical Genetics and Genomics (ACMG) guidelines (e.g., P for Pathogenic). Classification Criteria According to ACMG: Specific ACMG criteria codes used to classify the variant (e.g., PP3, BP5). HGMD/ClinVar/LOVD/Other: Databases where the variant is registered (HGMD: Human Gene Mutation Database, ClinVar, LOVD: Leiden Open Variation Database). Disease (OMIM): The disease associated with the variant according to the Online Mendelian Inheritance in Man (OMIM) database. Mode of Inheritance: Describes how the genetic variant is inherited (e.g., AR for Autosomal Recessive, AD for Autosomal Dominant).

Gene	Identified Variant		Zygosity	Genotype Notation According to HGVS v.15.11 (cDNA)	Allele Frequency in gnomAD (%)	Allele Frequency in POLGENOM (%)	ACMG Classification	Classification Criteria According to ACMG	HGMD/ ClinVar/ LOVD/ Other	Disease (OMIM)	Mode of Inheritance
	cDNA Notation	Protein Notation									
DYSF	c.863dupA	p.(Asp288GlufsTer40)	hom.	NM_003494.3: c.[863dup];[(863dup)]	-	-	P	ND	HGMD:DM LOVD:+/- UMD:DC	Miyoshi muscular dystrophy 1/ Muscular dystrophy, limb-girdle, autosomal recessive 2/ Myopathy, distal, with anterior tibial onset	AR
MYBPC3	c.814C>T	p.(Arg272Cys)	het.	LRG_386t1: c.[814C>T];[=]	0.0043	-		PP3, PP5, BP5	HGMD:DM Cvar:US LOVD:?/-	Cardiomyopathy, dilated, 1MM/ Left ventricular noncompaction 10 Cardiomyopathy, hypertrophic, 4	AD AD, AR
OPTN	c.1154A>T	p.(Lys385lle)	het.	NM_021980.4: c.[1154A>T];[=]	-	-		PM2, PP3, BP2	Not registered	Amyotrophic lateral sclerosis 12/ Glaucoma 1, open angle, E	- AD

Additionally, laboratory tests repeatedly showed significantly elevated levels of creatine kinase, with high values correlating with the clinical picture of the disease (3492 U/L) (Table 4).

**Table 4:** Laboratory data. H: High; ASPAT: Aspartate Aminotransferase; ALAT: Alanine Aminotransferase.

Parameter	Patient Value	Unit	Reference Range
ASPAT	72.4	U/L	(0.0-37.0) H
ALAT	101.9	U/L	(0.0-40.0) H
Creatine kinase	3491.6	U/L	(29.0-190.0) H

**Figure 1:**
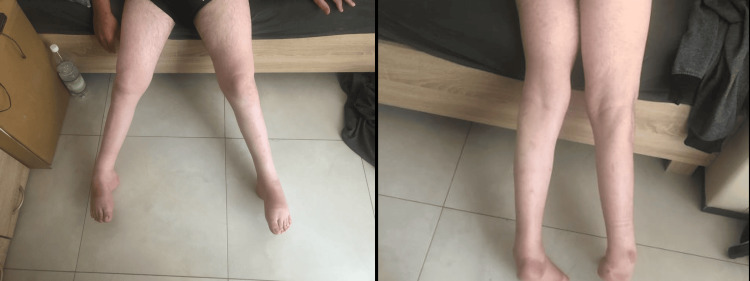
The calf's morphology, which shows significant atrophy, suggests a case of Miyoshi myopathy.

**Figure 2:**
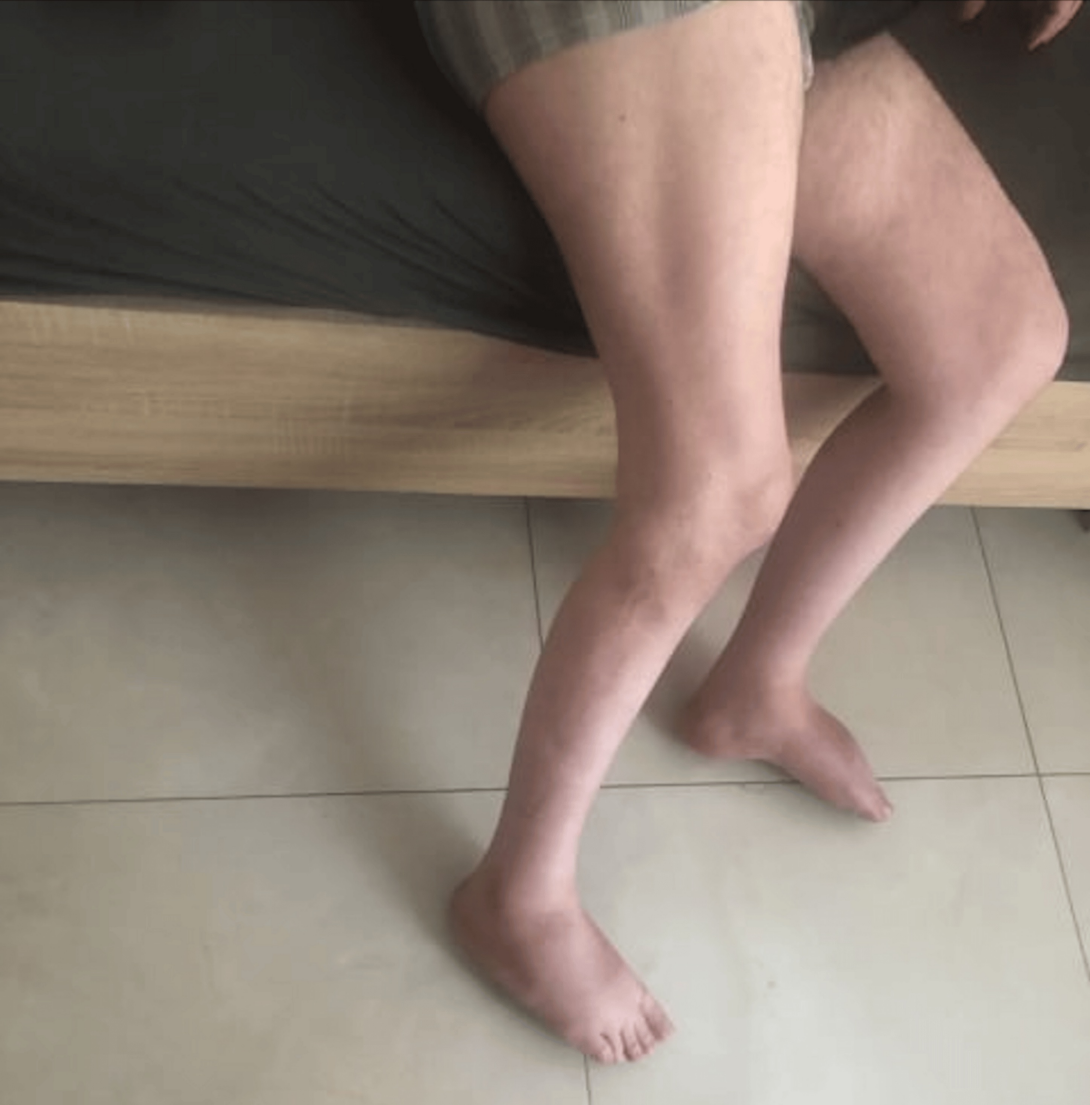
Marked atrophy of calf muscles.

Photographs of the patient's lower limbs (Figures 1-2) show significant muscle atrophy in the calves, consistent with the observed clinical symptoms.

Considering the overall clinical presentation, along with laboratory and electrophysiological findings, the patient was diagnosed with dysferlinopathy with a MM phenotype.

Due to the genetic nature of the condition, no curative treatment is available. The cornerstone of the patient's management comprises rehabilitation, physiotherapy sessions, and home-based exercises. These therapeutic approaches are designed to preserve muscle strength and function, slow the progression of symptoms, and enhance the patient's quality of life. The patient's response to physiotherapy and rehabilitation has been monitored on a regular basis. Despite the progressive nature of dysferlinopathy, these interventions have been effective in managing symptoms and maintaining a certain level of mobility and independence. The patient remains actively engaged in a personalized home exercise program tailored to his capabilities and needs. Regular follow-up is conducted to assess disease progression and response to treatment. Periodic evaluations include clinical assessments, electrophysiological studies, and laboratory tests to monitor muscle function and creatine kinase levels. Modifications to the rehabilitation program are made based on these assessments to optimize care and address emerging challenges associated with his condition.

## Discussion

The history of distal myopathies, including MM, is marked by significant milestones that have enhanced our understanding of these diseases. In the mid-20th century, Elisabeth Welander was among the first to describe distal myopathy as a distinct clinical entity [7]. She documented cases within 72 Swedish families, characterized by weakness in small hand muscles and autosomal dominant inheritance. This work laid the groundwork for further research into distal myopathies.

The first distinct cases of MM were reported by Miyoshi K et al. in 1977 [2]. This team identified MM as a condition characterized by significantly elevated levels of CK and initial weakness in the calf muscles. Following this publication, cases of MM began to emerge worldwide, highlighting its global presence.

In the 1990s, researchers identified specific genetic mutations associated with various distal myopathies. A pivotal discovery was the identification of mutations in the dysferlin gene (DYSF) linked to MM by Liu J et al. in 1998. These mutations are responsible not only for MM but also for other related muscular dystrophies, such as Limb-Girdle Muscular Dystrophy type 2B (LGMD2B) [8]. This discovery significantly advanced our understanding of the molecular basis of these muscular dystrophies and opened new diagnostic and therapeutic avenues.

As of 1996, there were only 60-70 reported cases of MM in the literature [9]. Over the past 28 years, the detection of this condition has significantly increased.

Approximately 1 in 440,000 individuals are affected with MM in Japan [10]. The typical age of onset for MM is between 15 and 30 years, with a median onset at 19 years. There is no significant difference in prevalence between sexes [11].

The initial clinical symptoms of MM typically include muscle weakness in the lower limbs, primarily affecting the calf muscles. Patients often report difficulties with activities such as climbing stairs, standing on tiptoes, and jumping. As the disease progresses, muscle weakness and atrophy spread upward to the thigh and buttock muscles, and in later stages, may involve the shoulder and arm muscles [3]. Elevated CK levels, often exceeding the upper limit of normal by several folds, are a significant marker of the disease [4]. Our patient presented with specific clinical symptoms as described above.

Diagnosis is based on the characteristic clinical presentation, laboratory, and genetic test results. Imaging studies, such as MRI, can help assess muscle condition and identify atrophy. EMG can distinguish myopathy from other neuromuscular diseases. The muscle biopsy can reveal characteristic changes such as muscle fiber atrophy and inflammatory cells [12].

The EMG examination performed revealed massive axonal damage to the motor fibers of both peroneal nerves in our patient. The genetic test results revealed the presence of a known pathogenic variant in a homozygous state. The probe of the biopsy was performed, but in cases of total muscle atrophy, the cores collected were not representative.

Currently, there is no cure for MM, and treatment focuses on symptom management and enhancing the quality of life. Regular physical and occupational therapy helps maintain muscle strength and flexibility, while assistive devices like braces and wheelchairs support mobility as the disease progresses. Pain management and strategies to cope with muscle fatigue are crucial for daily living. Genetic counseling is recommended for patients and their families to understand the inheritance patterns and family planning implications. Regular monitoring by healthcare providers is essential to adjust treatments based on disease progression. Participation in clinical trials is encouraged to access new experimental treatments and support research efforts. Recent developments in gene and cell therapies, including CRISPR-Cas9 and stem cell research, have shown promising results [13].

MM shares several similarities with CMT, which can complicate diagnosis. Both conditions initially present with muscle weakness in the lower limbs, with MM primarily affecting the calves and CMT affecting the feet and lower legs. Both diseases are progressive and involve additional muscle groups over time, leading to mobility issues. Elevated CK levels are characteristic of MM and less common in CMT. Both diseases have genetic underpinnings, with MM being autosomal recessive due to mutations in the DYSF gene, while CMT has various inheritance patterns and can result from mutations in multiple genes. Despite these similarities, differences in etiology, symptoms, and specific genetic markers help distinguish between the two conditions [3,14].

## Conclusions

The history and current understanding of MM illustrate the importance of genetic and medical advancements in diagnosing and treating rare diseases. Substantial progress has been made from the initial clinical descriptions to the discovery of dysferlin gene mutations. Ongoing research, particularly in gene and cell therapies, continues to offer hope for more effective treatments and an improved quality of life for patients with MM.

To the best of our knowledge, this is one of the first documented cases of a Polish patient diagnosed with MM. The objective of this study is to enhance physician awareness, thereby improving the recognition and diagnosis of this condition. Suspicion of Miyoshi distal muscular dystrophy should be considered in patients with significantly elevated serum CK activity and distal weakness of the lower limbs.
